# Improving learning and confidence through small group, structured otoscopy teaching: a prospective interventional study

**DOI:** 10.1186/s40463-017-0249-4

**Published:** 2017-12-28

**Authors:** Peng You, Saad Chahine, Murad Husein

**Affiliations:** 1 Department of Otolaryngology-Head and Neck Surgery, London Health Science Centre, Schulich School of Medicine & Dentistry, Western University, London, Canada; 2 Department of Medicine and Faculty of Education, Centre for Education Research & Innovation, Schulich School of Medicine & Dentistry, Western University, London, Canada

**Keywords:** Otology, Otoscopy, Medical education, Skills, Training, Knowledge

## Abstract

**Background:**

Otologic diseases are common and associated with significant health care costs. While accurate diagnosis relies on physical exam, existing studies have highlighted a lack of comfort among trainees with regards to otoscopy. As such, dedicated otoscopy teaching time was incorporated into the undergraduate medical curriculum in the form of a small group teaching session. In this study, we aimed to examine the effect of a small-group, structured teaching session on medical students’ confidence with and learning of otoscopic examination.

**Methods:**

Using a prospective study design, an otolaryngologist delivered an one-hour, small group workshop to medical learners. The workshop included introduction and demonstration of otoscopy and pneumatic otoscopy followed by practice with peer feedback. A survey exploring students’ confidence with otoscopy and recall of anatomical landmarks was distributed before(T1), immediately after(T2), and 1 month following the session(T3).

**Results:**

One hundred and twenty five learners participated from February 2016 to February 2017. Forty nine participants with complete data over T1-T3 demonstrated significant improvement over time in confidence (*Wilk’s lambda = .09, F(2,48) = 253.31 p < .001, η* ^*2*^ *= .91)* and learning (*Wilk’s lambda = 0.34, F(2,47) = 24.87 p < .001, η* ^*2*^ *= .66*).

**Conclusions:**

A small-group, structured teaching session had positive effects on students’ confidence with otoscopy and identification of otologic landmarks. Dedicated otoscopy teaching sessions may be a beneficial addition to the undergraduate medical curriculum.

## Background

Otologic diseases are common and associated with significant healthcare burden [1, 2]. Otopathology has been estimated to account for approximately 4 billion dollars a year in health care costs in the United States [3]. Relatively heavy health-care utilization is also seen in Canada, with the most common otologic condition seen in general practice being otitis media [4]. Given its variable presenting symptoms, accurate diagnosis relies on physical exam [5, 6], requiring an accurate and effective use of the otoscope and ability to examine the tympanic membrane [2].

Existing studies have highlighted a lack of comfort among trainees and practitioners with otoscopic examination [6, 7]. Uncertainty is often associated with overdiagnosis of otologic disease such as acute otitis media [8]. This, in turn, can give rise to the inappropriate use of antimicrobials and unnecessary specialist referrals [9, 10].

To date, various authors have cautioned against the under-representation of otolaryngology within the undergraduate medical curriculum [8, 11–13]. Efforts to improve otoscopy education have included Web-based teaching [14], endoscopic demonstrations [15], and high-fidelity simulators [13, 16]. While these education models have demonstrated some positive effects, they may not be readily assessable nor routinely incorporated within medical education. To improve trainee competency in otologic condition diagnosis and management, instructors have incorporated dedicated time for otoscopy teaching into the clerkship curriculum at Western University.

The purpose of this study was to examine the effect of a hands-on, small group otoscopy teaching session for medical students. Specifically, we aimed to evaluate its effect on students’ confidence with otoscopy in addition to learners’ recall of relevant anatomic landmarks.

## Methods

A prospective interventional study, utilizing self-report surveys, was conducted between February 2016 and February 2017. Participants included third-year medical students (clinical clerks) who participated in a dedicated otoscopy teaching session which was incorporated into the undergraduate medical curriculum as a small group teaching session. Participation in the study was voluntary and written informed consent was obtained. This study was approved by Western University Research Ethics Boards (file number 107347).

### Teaching session

A one-hour workshop was hosted by a fellowship trained otolaryngologist who was blinded to survey participation. The objectives of the session were to 1) teach relevant anatomy, 2) introduce appropriate otoscopy and pneumatic otoscopy technique, and 3) consolidate teaching through hands-on practice with immediate feedback.

First, didactic teaching introduced learners to key anatomic landmarks [2] and their clinical relevance through pictorial examples of normal and abnormal tympanic membrane. Second, the instructor reviewed proper technique in holding an otoscope and pinna as well as the use of pneumatic otoscopy. Participants were also advised on the choice of speculum size and how best to avoid patient discomfort during the exam (i.e., placing the speculum lateral to the hair and non-hair-bearing junction). Otoscopic examination of volunteer participants helped learner gain an understanding of otologic landmarks and a normal pneumatic otoscopy exam. Finally, participants were divided into pairs or groups of three, and practiced otoscopy/pneumatic otoscopy exams on each other under staff supervision. Learners received immediate, informal feedback regarding their techniques from peers and the facilitator for comfort and technique, respectively. This allowed the learner to adjust their technique until an adequate view of the tympanic membrane was attained in a way that did not cause discomfort.

### Questionnaire

**Fig. 1:**
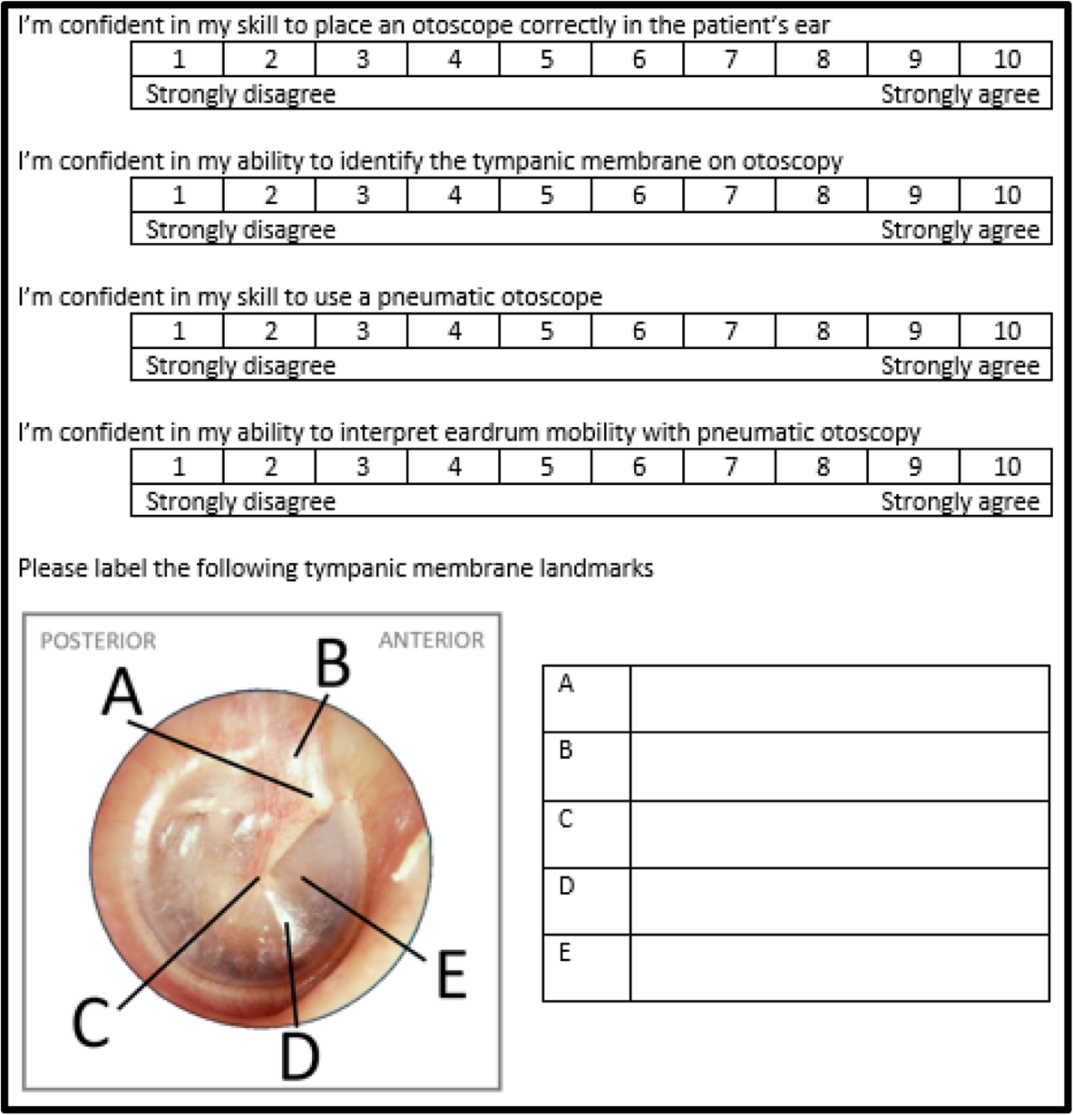
Core survey questions assessing confidence with otoscopy and learning of anatomic landmarks. Answer key for anatomic landmarks: **a** lateral process of malleus, **b** pars flaccida, **c** umbo, **d** light reflex, and **e** pars tensa

Participants were surveyed at three time points with questions designed to assess confidence with otoscopy and learning of anatomic landmarks [Fig. 1]. The tympanic membrane landmarks being evaluated are visible in a normal ear [2] and include: the lateral process of malleus, pars flaccida, umbo, light reflex, and pars tensa. Surveys were distributed prior to and immediately following the teaching session. A follow-up survey was sent 1 month following the teaching session through e-mail. Additional items included in the follow-up survey included the following binary questions: “Was this session useful?”, “Would you recommend the session to incoming clerks?”, and “Since the session, have you had the opportunity to use an otoscopy clinically?”. Two additional reminder e-mails were sent to non-respondents at 1 and 2 weeks to maximize the number of responses.

### Statistical analysis

Learner’s self-reported confidence was assessed using a ten-point Likert scale, ranging from 1 (*strongly disagree*) to 10 (*strongly agree*) (see Fig. 1). To capture partial learning, answers of anatomic landmarks were assigned a score from 1 to 4 (1-no attempt, 2-attempt but incorrect, 3-answer was that of a nearby structure, 4-correct answer). Descriptive statistic and reliability analysis were conducted as part of the development processes for both scales. Total scores were calculated for the anatomic landmark items and confidence items were averaged. Paired *t*-tests with 95% confidence intervals and Bonferroni corrections were used to evaluate the magnitude of pre-post change. For participants who completed the one-month follow-up survey, repeated measures ANOVA was used to evaluate the change across the three time points (T1, T2, T3). All of the analyses were conducted using SPSS version 24 [17].

## Results

Eighteen teaching sessions were conducted with an attendance of 7-8 learners per session. Of the learners, 125 of 143 (87%) went on to participate in the study, completing the pre- and post-session survey. Comparing pre- and post-session survey results, the self-reported confidence of otoscopy and pneumatic otoscopy improved significantly (3.12, *p* < 0.001). Similarly, participants’ scores increased for identification of tympanic membrane landmarks (5.96, *p* < 0.001) [Table 1]. The accompanied Cohen’s d value was 2.98 for confidence and 1.4 for learning, demonstrating a large effect size.

**Table 1:** Self-reported confidence and anatomy identification pre- and post- teaching session

	Pre-session (m/sd)	Post-session (m/sd)	Paired Samples t-test
Confidence	4.34 (1.12)	7.47 (1.06)	p < 0.001
Learning	9.86 (4.00)	15.82 (3.67)	p < 0.001

*m* mean, *sd* standard deviation

Forty-nine participants completed the 1 month follow up survey (response rate: 39%). Cronbach’s alpha for the anatomic landmarks scale (T1:0.82, *n* = 125; T2:0.70, *n* = 125; T3:0.77, *n* = 49) and confidence scale (T1:0.63, *n* = 125; T2:0.75, *n* = 125; T3:0.79, *n* = 49) showed acceptable internal consistency of the two scales. On follow up survey, 100% of participants found the teaching session to be helpful and would recommend the session for future students. Moreover, 96% of participants reported having used otoscopy in the 1 month following the teaching session.

**Fig. 2:**
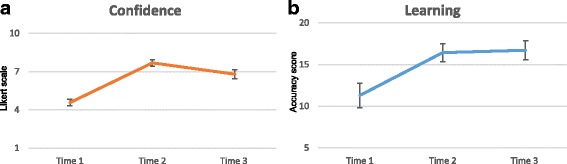
Graphical representation of (**a**) confidence and (**b**) learning as measured by pre-session (Time 1), post-session (Time 2), and 1 month follow up (Time 3) survey results. Error bars represent the 95% confidence interval

Data of participants who completed the one-month follow up survey were analyzed. The results showed improvement in confidence and learning following the teaching session compared to pre-session values. The one-way within-subject ANOVA was used to compare the means between the pre-session survey (T1), post-session survey (T2), and one-month follow-up survey (T3). For learner confidence, the ANOVA was significant (*Wilk’s lambda = .09, F(2,48) = 253.31 p < .001, η* ^*2*^ *= .91)* [Fig. 2a] and the Holm’s sequential Bonferroni procedure for pairwise comparisons showed significant (*p* < 0.001) differences between T1 (m = 4.46, sd = 0.89), T2 (m = 7.67, sd = 0.85), and T3 (m = 6.78, sd = 1.23). Mean confidence decreased between T2 and T3.

Similarly, the ANOVA for student learning was significant (*Wilk’s lambda = 0.34, F(2,47) = 24.87 p < .001, η* ^*2*^ *= .66*) [Fig. 2b], and follow up pairwise comparisons were significant (*p* < 0.001) between T1 (m = 11.29, sd = 4.71) and T2 (m = 16.43, sd = 3.59) as well as T1 and T3 (m = 16.71, sd = 3.69). There was no statistical difference between T2 and T3 (*p* = 1.00). In other words, as confidence decreased over time, learning at one-month remained at post-session levels.

## Discussion

While otoscopy and pneumatic otoscopy remain key tools in diagnosing otologic conditions [2, 3], these skills are difficult to teach. The tympanic membrane is relatively inaccessible which makes the objective assessment of otoscopy challenging. Furthermore, learners do not often receive feedback on their techniques. This difficulty may explain why the comfort level for otoscopy is low among medical trainee and clinicians of various disciplines [18, 19]. It also encourages educators to change the way otoscopy teaching is currently being delivered [8].

To address this need, dedicated time with an otolaryngologist was allocated to teach students otoscopy during the medical curriculum. Consistent with the published literature, our participants were not very confident with otoscopy prior to the teaching sessions [Table 1]. Our results also showed otoscopy is a commonly used clinical skill with 96% of participants reported using otoscopy in a clinical setting in the follow-up survey. The positive effects that our one-hour teaching session had on learners’ confidence and familiarity with anatomic landmarks was encouraging. The format of the didactic teaching, in addition to hands-on training with cooperative peers, and facilitated by an otolaryngologist who was available for immediate feedback, was shown to be well-received.

Education innovations have led to an increased effort to incorporate simulation in medical education [12]. The same is also true for otoscopy. Web-based (OtoTrain) and high fidelity simulator such as OtoSim have been shown to have a positive effect on learners’ confidence [13, 20]. However, these studies have thus far only surveyed participant response immediately following the intervention. In our study, participants showed a boost in confidence levels following the session as well as 1 month later when compared to pre-session measures, although there was a decline with time. Interestingly, by comparison, improved familiarity with anatomic landmarks was sustained. This suggests the possible need for repeat sessions to renew learners’ confidence.

The survey applied in this study has not been previously published, as a standardized tool for evaluation of otoscopy learning does not currently exist. As such, statistical analysis was used to examine the internal consistency of our assessment tool [21]. Cronbach’s alpha was calculated for the two variables assessed in the survey (self-reported confidence and labelling of anatomic landmarks). The alpha values approximate 0.7, which corresponds to acceptable internal consistency values, demonstrating that the scales used are reliable [22]. In studies with multiple time points, test familiarity/exposure is a limitation in measuring true change. Although our internal consistencies were high and stable across the three time points, interpretation of the results ought to take into consideration potential test-retest effects.

With regards to assessment of learning in the form of anatomic structures, the landmarks chosen are those that should be visible in a normal ear [2]. Learners’ familiarity with these key sites is crucial for their ability to assess the presence of otopathology. We aimed to capture partial learning by assigning answers a score of 1 to 4. In this way, we hoped to better represent the effect of the session. The improved familiarity with anatomic landmarks was complemented by results of increased confidence.

Limitations of this study include participation rate and the self-report nature of the survey. While 87% of the learners participated in the pre- and post-session survey, only 39% of participants completed the follow-up survey at 1 month despite reminder emails. The average individual survey response rate for academic research was found to be 52.7% with a standard deviation of 20.4 [23]. Though our response rate at 1 month falls within the standard deviation, the findings are still vulnerable to non-response bias. With regards to the self-assessment aspect of the results, confidence is inherently a self-reported measure and in turn prone to reporter bias [24]. Furthermore, it is uncertain if comfort and confidence extrapolate to improvement in diagnostic ability. One study proposed that confidence level of practicing physicians may be disproportional to their skill set [25]. On the other hand, various literature in social sciences has identified self-confidence as important in the acquisition of skills and beneficial to performance [26–28]. In our study, we showed the teaching session to have a positive effect on confidence as well as improvement in identification of anatomic landmarks, both of which are important components of an otoscopic examination. It remains unclear how much each component within the multi-component nature of our intervention (didactic teaching, hands-on demonstration, small group learning, and immediate feedback) contributed to the confidence and knowledge.

Our study was also limited by the absence of control group and otopathology. The teaching session described is a new addition to the undergraduate medical curriculum at our institution. Hence, we arrived at the present single arm pre-post study design. Future research may incorporate didactic teaching and/or simulation as comparison arm. Furthermore, as the participants were practicing with their peers, they were essentially observing healthy, adult ears. This also precluded the examination of challenging canal or pediatric ear. To help assess the ability to identify anatomic landmarks in a variety of ears, future iterations can use different pictures of both normal and abnormal otoscopic exam. One advantage of high fidelity simulators in this setting is its ability to present pathology and assess diagnostic ability. Recent studies have demonstrated positive effects using simulation with a sustained improvement of diagnostic accuracy at 3 months follow up [29, 30]. Unfortunately, the drawback to otology simulators is that they are not yet readily incorporated into the medical curriculum.

Nevertheless, in lieu of any simulators, we showed that a facilitated teaching session by an otolaryngologist in a small group format can have positive effects. Our results can help educators devise an effective way to teach otoscopic examination in a manner that is well accepted by the learners. It remains to be seen if a similar outcome can be replicated by experienced facilitators from other fields other than otolaryngology.

## Conclusions

Small group, structured teaching sessions are an accessible and effective way of teaching otoscopy with positive effects on learner confidence and familiarity with key anatomy. The sessions were well received and point to the valuable role of dedicated otology teaching sessions within undergraduate medical education.

## Abbreviations

CI: Confidence interval
m: Mean
sd: Standard deviation
